# Online support groups for family caregivers: A qualitative exploration of social support and engagement

**DOI:** 10.1111/bjhp.12764

**Published:** 2024-11-07

**Authors:** Rosemary Daynes‐Kearney, Stephen Gallagher

**Affiliations:** ^1^ Department of Psychology, Centre for Social Issues Research, Study of Anxiety, Stress and Health Laboratory University of Limerick Limerick Ireland; ^2^ Health Research Institute University of Limerick Limerick Ireland

**Keywords:** engagement, Facebook, family caregivers, framework for engagement, NVivo, Online support groups, qualitative analysis, social support

## Abstract

**Background:**

This qualitative research explored family caregivers’ engagement and social support in a single online support group (OSG). It sought to answer two research questions: Q1: Was social support evident in the OSG? If so what types and how did these types relate to engagement? Q2: Were elements from the Context, Content and Delivery conceptual framework evident in the OSG? If so, what were the key elements of this group?

**Methods:**

Eighteen semi‐structured interviews were carried out with members of a family caregiver OSG in Ireland. Data were analysed using deductive qualitative analysis with a codebook created from one typology of social support and the Context, Content and Delivery conceptual framework for engagement in web‐based technologies.

**Findings:**

For Q1, all types of social support were generated in the OSG. All had a positive impact on engagement. Informational support (17/18 respondents) and emotional support (15/18 respondents) were the highest support experienced by the group. For Q2, we found evidence for 11 key elements of the conceptual framework. Myriad sub‐elements had positive, negative and mixed impacts on engagement. These elements generally related to positive developments of social support by the respondents.

**Conclusion:**

Drawing together the findings, we present a new framework, the Journey of Engagement and Support in Online Support Groups for Family Caregivers. This maps the stages for engagement and support in an OSG and can be used by practitioners for running OSG and researchers to generate testable hypotheses about the relationship between social support and engagement.

## INTRODUCTION

Social support is a multidimensional concept that includes different facets of support such as structure and functional aspects including information, emotional and tangible support (House et al., 1985, del‐Pino‐Casado et al., 2022). Social support can be defined as ‘social interactions or relationships that provide individuals with actual assistance or that embed individuals within a social system believed to provide love, caring, or a sense of attachment to a valued social group or dyad’ (Hobfall, 1988, p. 121). It has consistently been identified as an important factor to mitigate some of the negative impacts of family caregiving, such as caregiver burden (Rodakowski et al., 2012), social isolation (Daynes‐Kearney & Gallagher 2023a), cardiovascular risk (Gallagher & Whiteley, 2012) as well as promoting positive elements such as psychological resilience (Donnellan et al., 2017; Lök & Bademli, 2021). While historically, social support was developed in face‐to‐face settings, the advent of the internet and social media has seen family caregivers use online settings such as Facebook groups (Newman et al., 2019), web‐hosted groups and videoconferencing (Damianakis et al., 2016) for social support.

A recent scoping review of online support groups (OSGs) demonstrates that they are widely used by family caregivers as a means of accessing support (Daynes‐Kearney & Gallagher, 2023b). There is a large body of research into OSG and health‐related online support communities. These examine the nature and impact of online social support across diverse patient groups, such as breast cancer patients (Setoyama et al., 2011), patients with Complex Regional Pain Syndrome (Smedley et al., 2015) and patients with neuromuscular disorders (Meade et al., 2018). Some research has focused on understanding if and how online communities can contribute to the well‐being of individuals, with general findings demonstrating OSG provide emotional and informational support, reduce feelings of isolation and are places to share experiences (e.g. Elwell et al., 2010; Rowlands et al., 2023; Setoyama et al., 2011). Investigations into the psychosocial mechanisms that underpin OSG have revealed that the shared space was a central component where people could share difficulties such as getting recognition (Meade et al., 2018) and share universality of experiences (Setoyama et al., 2011). Additionally, empowering processes such as connecting to others, helping others, finding recognition and understanding (Mo & Coulson, 2014) and using self‐disclosure to elicit support (McKechnie et al, 2014; Wang et al., 2022) were important factors.

While a smaller area of research than that relating to patients, there is an increasing body of work researching OSGs for family caregivers. This research demonstrates that caregivers feel positive about web‐based interventions (Wasilewski et al., 2019). Similar to research with other cohorts, they have positive benefits for caregivers, such as relieving social isolation (Greenwood et al., 2013), increased advocacy skills (O'Donnell et al., 2024) and benefits for enhancing self‐efficacy (Pagán‐Ortiz et al., 2014). However, research has also shown inconsistency in results. For example, one systematic review (Dam et al., 2016) found while qualitative outcomes indicated reduced social isolation and increased emotional support (e.g. Greenwood et al., 2013), quantitative outcomes were not significant (e.g. Charlesworth et al., 2008) and concluded that there was insufficient evidence to determine whether changes in social support are a mediating factor in benefits to well‐being. Therefore, while there are many positive effects of OSG reported, questions remain about the types of social support generated in OSG and how the groups are experienced by group members.

It is also widely posited that engagement by members in OSG is vital to their success (e.g. Washington et al., 2020). There is not one agreed definition of engagement in OSGs or communities. Nevertheless, common characteristics of engagement include the degree of participation such as posting, responding and interacting with other members (Benson et al., 2020; Jeong et al., 2018), or connecting with others, sharing experiences and seeking and providing support (Meade et al., 2018; Mo & Coulson, 2014), while Perski et al. (2017, p. 258) define engagement with web‐based interventions as ‘(1) the extent (e.g., amount, frequency, duration, depth) of usage and (2) a subjective experience characterized by attention, interest and affect’ that is a combination of user behaviour and user experience.

Differentiations have been made between passive engagement which is primarily reading posts without commenting and active engagement of reading, posting and commenting on content (e.g. Coulson, 2015; Setoyama et al., 2011; Trail et al., 2020; van Uden‐Kraan et al., 2008; Vaughan et al., 2018). Furthermore, questions have been posed around the relationship between engagement in OSG and social support, including what is meant by engagement in OSG and what elements of engagement are important to successfully generate social support in online group settings (Daynes‐Kearney & Gallagher, 2023b).

Some research on patient groups and engagement in OSG and online communities found that passive participants were involved for shorter periods and visited the groups less frequently than active participants (Coulson, 2015; van Uden‐Kraan et al., 2008). While both passive and active participants received similar levels of advice or informational support (Vaughen et al., 2018), active participants experienced higher levels of emotional support and were freer with emotional expression (Setoyama et al., 2011). Research with patient groups has also identified some of the barriers to engagement for participants of OSG (Coulson, 2015). These barriers were consistent across family caregiver groups, with time constraints due to caregiving commitments as an additional significant factor (Vaughan et al., 2018; Washington et al., 2020).

However, despite the existing bodywork on OSG for family caregivers, there is no one framework for understanding engagement in OSG for family caregivers and how this relates to social support in that context. Understanding these engagement dynamics is crucial to optimizing the effectiveness of OSGs for family caregivers who will provide insights into how OSGs can be better designed and run to meet their needs.

Therefore, this study investigates whether social support was experienced by members of one OSG for family caregivers in Ireland and what elements were important for participants to encourage or discourage their engagement in the OSG. As such, this study sought to answer two research questions:

Was social support evident in the OSG and if so what types? How did these types of social support relate to engagement in the group?

Were elements from the Context, Content and Delivery conceptual framework (Perski et al. 2017) evident in the OSG? If so, what were the key elements of this group?

## METHODOLOGY

The first author conducted semi‐structured interviews with 18 members of Care Alliance Ireland Online Support Group (www.carealliance.ie) in Ireland. It is run via a private Facebook group with a range of activities via videoconferencing tool Zoom also available to members. The OSG has received annual funding from the Irish Health Service Executive (HSE) since 2021. At the time of the research (November 2021–February 2022), the group membership was over 3000, this has now grown to over 8000.

The sample was self‐selecting existing members who responded to internal group posts, information sheets and a live‐streamed information session developed by Author 1 and the Care Alliance Ireland team. Of 32 people who expressed an interest in participating, 18 decided to continue the process to the full interview. The most common reason given for someone not proceeding further was the time commitment required. All participants were interviewed online via MS Teams over a period of 4 months. Interviews ranged between 35 and 75 min with an average of 55 min. As a mark of appreciation, each participant was sent a €25 gift voucher following the interview. All participants were invited to review the interview transcripts; however, none of the participants chose to do so.

Information power (Malterud et al., 2016) was determined during the recruitment process. All interviews were recorded and transcribed using the automated transcription service by MS Teams which was subsequently quality checked by the first author. The project was also registered on Open Science Framework [OSF Registries | Online Support Groups for Family Caregivers ‐ Qualitative Study]. The project received ethical approval from the institute's ethical review board [Reference Number: 2021_11_07_EHS]. This study was conducted from a critical realist stance and both researchers are current family carers. A more detailed description of the context, sample selection and data collection are available in Daynes‐Kearney and Gallagher (2023a).

### Data analysis

The transcripts were transferred to NVivo (Version 1.7.1(1534): QSR International). Data were analysed using a process of deductive qualitative analysis. A codebook was created from Cutrona and Suhr (1994) typology of social support and Perski et al. (2017) framework for engagement in web‐based technologies (Tables 1a and 2a in Appendix S1). Data were analysed through repeated coding cycles, which enabled refinement of the elements and space was given for inductive codes to be discovered. Researchers used a reflexive approach with regular meetings to facilitate both the discussion of the data and values, beliefs and attitudes that may be influencing the interpretation (Saldaña, 2021). Trustworthiness in the findings was also developed through ongoing engagement with the Care Alliance Ireland team, including co‐presenting the preliminary findings at a global conference and sharing pre‐publication findings, and regular updates via posts to the group. Internal peer review was engaged in during the entire research process.

These ongoing discussions were used to refine the coding to ensure the robustness of the interpretation process. Specifically, in relation to Perski et al. (2017) framework, consideration was given not just to whether a factor was mentioned during the interviews, but the context and impact of the factor. As such, when deciding about the key elements in relation to this OSG, the factor had to appear more than once, be related to this OSG and have an impact on support or engagement in this group. For example, although computer literacy was mentioned in one interview, the context was judged as related to factors outside the OSG and the impact was not related to engagement in this OSG. On other occasions, it was decided that a factor may sit as a subfactor to another factor. For example, although the group was provided with tutorials and guidance as to how to register for the group and to use different technologies, there were references in terms of professional support in the group. As such, it was decided that professional support is the key factor with actions such as providing tutorials as elements of this support.

Demographic information was tabulated along with information regarding the participants’ description of their level of engagement. This enabled a comparison of demographic features alongside information about their engagement in the group, which although not analysed using statistical testing, adds depth to the findings (Table 1, Table 1a in Appendix S2). The data analysis process demonstrated that social support was generated in the OSG and that many constructs from Perski et al. (2017) framework were identifiable in the group. These are presented below and illustrated using relevant verbatim quotes.

**TABLE 1:** Demographics of interview participants.

Gender	16 female, 2 male
Age range	39–77 (*M* = 52)
Caring relationship	10 parents, 3 spousal, 2 sibling/in law, 2 child 3 participants caring for more than one person
Geographic area	3 Dublin Urban, 4 Other Urban, 4 semi‐urban/semi‐rural, 6 rural
Frequency of engagement	10 several times daily, 3 daily, 4 frequently, 1 infrequently

## FINDINGS

The research participants were primarily women in a middle‐aged range (40–69). Two male carers participated in the research, which broadly aligns with the known gender breakdown of carers in this age group in Ireland (Family Carers Ireland, 2022). All participants identified as white, 16 identified as Irish only, and two identified as a mix of Irish and another European nationality. The research participants all engaged with the group frequently, with notifications coming through several times a day as content was posted. All engaged by reading the material in the group and commented back on posts, either textually or using emojis. The majority had posted into the group, and many took part in at least one activity. This indicates that the group of participants was well placed to discuss this OSG for insights into the research questions.

### Question 1: Was social support generated in the OSG and if so, what types? How did these types of social support relate to engagement in the group?

Overall, the general feedback about the group was that it was a welcoming space with words like ‘camaraderie’ and ‘friendliness’ used recurrently to describe the feeling of the group. Across 17 interviews, some or all the types of social support were reported (Table 2). Information support was the most described type of social support, with social network, tangible and emotional support also very highly represented.

**TABLE 2:** Types of social support present in the group.

Type of social support	Present	How presented	Impact on engagement
Information	17/18 (94%)	Helpful information on main page and comments Diversity of information High quality Comments could have mixed quality	Overall positive impact on engagement, although comments could have negative impact by people reading inaccurate information
Emotional	15/18 (83%)	Honest self‐disclosures Sharing positives and challenges Use of emojis and pictures Encouragement and empathic statements in response to posts	Positive impact on engagement People felt emotional burden was relieved, enjoyed sharing and supporting others
Esteem	8/18 (44%)	Affirmation to poster they were doing their best Affirmation that they were providing a valuable role Positive non‐judgemental responses to self‐disclosures Acknowledgement of valuable contribution of sharing knowledge and skills	Positive impact on engagement People felt valued and visible, safe and relieved of stigma and emotions such as guilt or failure
Social Network	13/18 (72%)	Sense of feeling connected to large group of people in similar situations Removal of geographical barriers to enable building connections with others Removal of time boundaries to enable creation of connections with others	Positive impact on engagement People had more social connections through the group, connected with people they wouldn't meet locally and at times that suited them
Tangible	13/18 (72%)	People received useful resources from the organization People received gifts from the organization Items were advertised and passed on through the group	Positive impact on engagement These resources directly and indirectly helped people in their caring role

Information seeking served as pathway for many to join the group. Participants found the group well organized with lots of information available in it from group members and the moderators themselves. Of note was that the information was a mix of general information and advice based on the experiences of group members and that people were happy to share their knowledge if they felt it would be of benefit (Table 2). The diversity of the group make‐up was noted as exposing the group members to knowledge and experiences from caregivers of diverse backgrounds that they would not have come across in groups that were condition specific. In particular, it was the commonality of experience rather than commonality of condition that was beneficial. Overall, respondents felt that the quality of the information was high. However, several noted the challenge of ensuring of maintaining accuracy of some information in the comments replying to questions, where some information posted may not have been factually correct. There was an acknowledgement that the moderators did close comments on posts where required.

The group was regarded as a safe space that people could use to honestly express how they were feeling. Emotional support was not just for when things were tough but also when there were things to celebrate as well (Table 2). This emotional sharing was received in a non‐judgemental manner by the groups; people were not told to cry or not to feel guilty but were allowed to experience those emotions as they were. Participants affirmed or were affirmed that they were doing a good job in their role as a carer. This has the impact of increasing self‐confidence and feelings of self‐worth (Table 2). Many of the participants expressed how being part of the group made them feel like they were part of something, that they had a voice and a place to break stigma around caring. The level of connection participants felt to others in the group ranged from reading of posts to others who felt they had developed strong friendships with individuals in the group (Table 2).

Surprisingly, there were prominent levels of tangible support present, delivered primarily through the support organization itself. This included sending out free books for both leisure reading and care‐related manuals, sending gifts such as power banks and seeds as well as setting up access to free counselling for several of the participants. Tangible support between participants was expressed by the passing on of items that were no longer required and putting up direct links such as car parking maps in response to questions that were asked in the group. Table 2 illustrates the types of social support generated and its impact on engagement, and Table 2a in Appendix S2 is an enhanced table with quotes from participants.

### Q2: Were factors from the Context, Content and Delivery Conceptual framework (Perski et al. (2017) evident in the OSG? If so, which were the key factors for this group?

Of the 35 elements included in Perski et al.'s (2017) framework, the analysis identified 11 that were key in this OSG setting (Table 3). Seven of these were existing, and four were new categories added during the analysis. For each main element, sub‐elements were identified and explored in how they impacted on participants’ engagement in the group. Four Contextual elements were found to be key to engagement. We reconstructed two elements (*Constraints to Physical Environment* and *Constraints to Time*) as one element due to their interconnected nature and added a new element, Existing Social Support. In relation to Content, one element from the Perski et al. (2017) framework was included in our analysis with two new elements of ‘*Activities*’ and ‘*Incentives for Engagement*’ created. For Delivery, four elements were found to be important. *Mode of Delivery* and *Ease of Use* and *Professional Support and Interactivity* were combined, and the analysis established three separate but related components that led to *Credibility*, these were *Safety, Privacy* and *Trust*.

**TABLE 3:** Elements identified as key to engagement in family caregivers OSG.

	Context	Content	Delivery
Perski et al. (2017) framework elements	Constraints to Physical Environment and Time^a^	Social Support Features	Mode of delivery and Ease of use^a^
	Expectations		Professional Support and Interactivity^a^
	Personal relevance		Tone
New elements	Existing social support	Activities	Credibility: Safety, Privacy, Trust
		Incentives for engagement	

^a^ Two separate elements in the Perski et al. (2017) framework that were combined to make one element in this data analysis.

### Context

Of the four contextual elements, *Constraints to Physical Environment and Time* had clear positive effects on engagement, while *Existing Social Support* had a negative effect Table 4 and Table 4a in Appendix S2 for enhanced table with quotes from participants). The remaining two elements, Expectations and Personal Relevance, could have positive or negative impacts depending on how the participant experienced the group.

**TABLE 4:** Contextual elements, engagement and social support in the group.

Elements	Sub‐elements	Impact on engagement	How related to social support
Constraints to physical environment and time	Group responded to COVID‐19 lockdowns and withdrawal of services	Positive— provided an outlet for carers when no others existed	Provided all types of social support
	Flexibility and availability at any time	Positive—could engage when able to	Facilitated easy provision and receipt of social support
Expectations	Clear understanding of group purpose	Mixed—maintains engagement if understood what group was about, but negative if unclear e.g. name support group can mean different things	Important for group maintenance for participants continue to avail of opportunities for support
	Experience congruent with expectations	Positive—influenced decision about getting involved and how deep involvement would be in group	Important for group maintenance for participants continue to avail of opportunities for support
Personal relevance	Group content and members were relevant to participant	Positive—Comfort with people in similar situations	Provided all types of social support
	Social comparison	Mixed—can discourage engagement if feel others need more support	Can reduce opportunities to provide or receive social support
Existing social support	Other places to get social support	Negative—if had other avenues affected frequency and type of engagement	Can reduce opportunities to provide or receive social support in the group

Many of the participants had joined the group in the COVID‐19 pandemic as traditional support and respite services were shut down. The element of flexibility and availability of the group was a key factor in supporting participants’ engagement in the group. In general, participants were clear about what the group was about and what it offered. There was a sign‐up process to join the group including agreeing to the group rules before administrators granted access to join. However, there was a mismatch between expectations and experience at times. This draws attention to the need for OSGs to have a clear explanation of what they mean by ‘online support group’ as this could mean different things to different people.

Participants stayed in the group because it was personally relevant to them. For some participants, engagement was influenced by whether they had other support channels that they could use. Some people had friends or family that they could turn to for support and this meant that they used the group less frequently and for less support intensive engagement. Others used other online support channels and were members of more than one OSG. For others, this group was their primary avenue to receive social support and they used the group to meet more of their needs. Several participants commented that the development of an online group to support family carers was overdue and that a group like this should have been set up before COVID‐19.

### Content

All three content features included in the analysis had positive and negative effects on engagement in the group (Table 5 and Table 5a in Appendix S2 for enhanced table with quotes from participants). Facebook as a platform has in‐built social support features which the group used to give and receive social support, such as text‐based posting, image‐based posting, the ability to pin posts and to set up tabs for information. A key enabler to engagement was the direct messaging (DM) feature. Participants used this to contact moderators privately to ask questions that they did not want to post on the main site, or to contact other members privately in response to questions they may have raised. The use of direct messaging with the moderators was successful because the moderators responded in a timely manner to the person. It also facilitated participants to request to post anonymously on the main group page, which was noted repeatedly as a way for people to access support that they normally would not.

**TABLE 5:** Content elements Engagement and Social Support in the group.

Elements	Sub‐elements	Impact on engagement	Related to social support
Social Support Features	Direct messaging	Positive—enabled participants to ask questions or post anonymously	Facilitated easy provision and receipt of social support
	Variety of communication features	Positive—could engage how most comfortable	Facilitated easy provision and receipt of social support
	Timely response by moderators	Positive—build trust with participant	Key for informational and emotional support
Activities	Variety of activities	Positive—something for everyone	Provides opportunities to develop social network support
	Developed in response to expressed need in the group	Positive	Important for group maintenance to continue to avail of opportunities for support
	No expectations on participant	Positive—relieved pressure on participants and encouraged repeat attendance	Important for group maintenance to continue to avail of opportunities for support
	Unclear how to participate	Negative—prevented from joining or continuing with group	Can reduce opportunities to provide or receive social support
	Had to participate in real time	Negative—not always suitable time for participants	Can reduce opportunities to provide or receive social support but important for social network support
	New members joining established groups	Negative—familiarity made it difficult for new people to take part in group	Can reduce opportunities to provide or receive social support
Incentives for engagement	Spot prizes, competitions, gifts and targeted events	Positive—Fun way to be involved and build community	Builds all types of support
	Immediacy of some incentives	Mixed—not everyone able to respond quickly due to caring responsibilities	Can reduce opportunities to provide or receive social support
	Personal messages and ‘nudges’	Positive—welcome and encourage participant in the group	Key for development of emotional, esteem and social network support
	Group getting bigger	Mixed—concern over how to keep personal touches and sense of community with large group	

A distinctive feature of this group was the programme of activities on offer for group members, including a book club, gardening club, quiz night, chair yoga and skills development for carers who wish to return to work. These activities were immensely popular and were seen as creating community and providing different avenues to all types of social support.

A recurring challenge expressed by participants was how to manage dominant members in these activities. This indicates that a clear process is required about how to bring new people into an established group. It also highlights the importance of moderators receiving training in group dynamics and group facilitation skills as well as being proficient in managing the technical aspects of the online group. Participants reported that the group was well moderated and spoke about various techniques such as spot prizes and personal ‘nudges’. These built community and encouraged engagement; however, concern was expressed about how to keep the sense of community as the group got bigger.

### Delivery

The use of Facebook as the mode of delivery was positive for all participants. It removed the geographical proximity preventing participants from availing of supports in their local community (Table 6 below and Table 6a in Appendix S2 for enhanced table with quotes from participants). The majority stated that they would not have found the group if it was not on Facebook as this was the only social media platform they used and the group coming up in their feed or in their recommendations was a central enabler of carers joining the group. Several acknowledged that Facebook may appeal to a certain age group so the group may be missing younger people who could use support. There was a general concern about carers finding the group if the group used a different mode of delivery than Facebook. This demonstrates the importance of social media in finding participants to use the group and that Facebook as a platform is a popular choice for carers of a certain age who may be excluded if OSGs are operated on different social media platforms or other online settings.

**TABLE 6:** Delivery elements, engagement and social support in the group.

Elements	Sub‐elements	Impact on engagement	How related to social support
Mode of delivery and ease of use	Use of Facebook	Positive—not geographically bound, easy to find, part of day‐to‐day life	Facilitated easy provision and receipt of social support
		Mixed—used by a certain age group	None found
	Use of Videoconferencing	Mixed—enabled face‐to‐face real time interactions but technology was a barrier to some	Key for development of social network support
Professional support and interactivity	Trained moderators	Positive—participants feel group is managed well	Facilitated easy provision and receipt of social support
	Two‐way flow of communication	Positive—builds relationships	Facilitated easy provision and receipt of social support
	Welcoming new members	Negative if new members not welcomed to group	Key for development of emotional and esteem support
	Responsiveness	Positive—participants feel that their input is important	Key for informational and emotional support
	Dominant members	Negative—can take over group and mean activities do not meet expectations	Can reduce opportunities to provide or receive social support
	Moderator biographies	Mixed—can help participants to know any caring background of moderators	Can contribute to social network support
Tone of group	Friendly and fun	Positive—fun way to be involved and build community	Important for group maintenance and opportunities for support
	Non‐judgemental	Positive—encourages people to share and seek support	Key for emotional and esteem support
	Modelling group rules and norms	Positive—welcome and encourage participant in the group	Important for group maintenance to continue to avail of opportunities for support
	Problems dealt with quickly and respectfully	Positive—people see group as positive place	Important for group maintenance to continue to avail of opportunities for support
Credibility	Safety created by group rules being maintained	Positive—people feel that group is safe to be part of and use group	Key for development of emotional and esteem support
	Privacy—what is said in the group stays in the group	Positive—encourages people to share things that they would not otherwise	Key for development of emotional and esteem support
	Privacy enabled by anonymous posting	Positive—encourages people to share things that they would not otherwise	Key for development of emotional and esteem support
	Trust developed by moderator consistency, high‐quality information, effective communication on decisions	Positive—participants understand process behind decision making	Key for development of emotional and esteem support

Although Facebook was the sole point of entry into the group, once members had joined there was an additional mode of delivery for the activities which was through videoconferencing. This group used the platform Zoom for the delivery of the activities which allowed the ‘face‐to‐face’ element of traditional support groups to be replicated. The activities required that participants be available at a certain time. In general, people who wanted to take part in the activities found the videoconferencing easy to use, and there was guidance from the moderating team if people did have difficulty accessing events. However, for some, the videoconferencing format was too overwhelming, while for others getting familiar with a new technology was too much to do when trying to manage so many other aspects of caring. The face‐to‐face elements of videoconferencing can present the same challenges as ‘in person’ groups, and there was an acknowledgement that managing groups and interactions could be more difficult in an online setting, in particular, managing dominant members and supporting new members to feel comfortable.

A key enabler to engagement was that the group was run by a professional organization with trained moderators. There was a two‐way flow of communication between group members and between group members and moderators. Moderators welcomed new members if possible and posted up fresh content, provided guidance on how to use the group and technologies and incentivized engagement by spot prizes, mini competitions, giveaways and activities which encouraged people to join in. The responsiveness of moderators to direct messages developed a level of trust and credibility (see below) which enables members to feel safe in the group. Activities were added on a frequent basis, often in response to expressed needs or interests that were emerging in the group. This was reflected as the moderators learning and evolving with the group.

The tone of the group and use of humorous and positive messages in posts and at the activities was commented on frequently by participants as making them want to go onto the group and check in. Participants expressed gratefulness for the many gifts that they received from the organization, which sometimes would come as a surprise. This added to the feeling of well‐being and esteem building experienced by people who had received the gifts and by others who saw the pictures and posts of thanks from those who had received the gifts. Participants commented that these gestures brought visibility to their role as a carer.

The credibility of the group was formed by three separate but related elements, safety, privacy and trust. Safety was created from the outset, with group rules that had to be signed prior to joining. The group moderators were responsible for ensuring that the group rules were maintained. They were also responsible for ensuring that a balance was reached between allowing people to express themselves freely while keeping the boundaries of the group. Participants commented that they felt safe in the group as they had seen rule breaches dealt with appropriately by the moderators.

Privacy was considered one of the most principal elements for the group to operate successfully. Respect for privacy was set out in the group rules, and participants were also mindful of the privacy of the people they were caring for as well as their own. Several observed that some people would post a lot of personal information that may be more than they themselves would feel comfortable with, but that it was a matter of personal responsibility about how much people wanted to post. The value of anonymous posting was noted by participants in providing a support option for people who would not feel comfortable with posting directly on the group. None of the participants liked to think of carers being isolated by themselves or under emotional stress and pressure and appreciated that even if they did not want to use this option themselves it was there for those who did.

Trust was established by participants observing the interactions with the group and seeing consistency in how the moderators operated in the group. Participants commented that questions were answered quickly and that while not all information in the comments could be stood over, there was a high level in the quality of information being provided by the moderators themselves. Trust was also established by the moderators being transparent with group members around decision making. For example, if a post was not approved for posting, the moderators would revert to the original poster and explain their decision. Moderators welcomed feedback and try to maintain an open channel of communication with participants. It was suggested that it would be helpful if members knew a bit of background about the moderators, with short bios being published, especially if the moderator was a carer or former carer.

## DISCUSSION

This study explored the types of social support generated in one OSG for family caregivers and how this related to engagement. Using the typology by Cutrona and Suhr (1994), all types of social support were identified, with informational support most reported, which is consistent with other research (e.g. Coulson & Buchanan, 2022). Research into social support in OSG has typically found that informational and emotional supports are the two most prevalent forms of social support (e.g. Mohd Roffeei et al., 2015; Wasilewski et al., 2019). Surprisingly, there were high levels of tangible support in the CAI OSG by the organization itself and by participants to other participants which presents a novel finding of this study. Weiss et al. (2013) suggests that existing typologies of social support may not fit adequately with OSG as social support may be conveyed differently in online settings. Our findings indicate that this existing typology can be used effectively in OSGs, but that careful attention is needed over how the different types of support are coded to ensure they are correctly accounted for.

Our study also used the framework proposed by Perski et al. (2017) to examine in greater detail key elements for engagement in this OSG. Our results broadly concurred with Perski et al.'s (2017) framework but has extended on that framework and added new knowledge by investigating the specific reasons for impacts on engagement. We identified 11 key elements and myriad sub‐elements that had positive, negative and mixed impacts on engagement. Of note was the role of contextual factors such as physical and time constraints and requirement for information that drove people to seek out OSGs, the different experiences of participants to different modes of delivery, and the importance of meaningful interaction by the moderators to incentivize and encourage engagement and support.

Washington et al. (2020) also used Perski et al.'s (2017) framework for engagement. They found that contextual factor of caregiving commitments was one factor that negatively influenced engagement and highlighted the important role of the facilitator ‘tagging’ individuals and individualized attention to encourage engagement. Similar again to our own results, this study and others have found that Facebook was a convenient method of delivery (Parker Oliver et al., 2014) but that the tone of the group can often be mixed, with some participants finding groups depressing (Washington et al., 2020). This finding differed considerably from the reported tone of the CAI group which was described as friendly and positive. This finding could be attributed to the active role of the facilitators in the group and is a possible area for further research. The importance of the role of group moderator must be noted and as has been found in other research (Deng et al., 2023); here they were central to initial engagement, continuous engagement but also key to developing the conditions for development of trust and safety within the group. Further, while our group did have a moderator, it has become increasingly important to provide training and support for OSG moderators (The Light Collective, 2020).

One concern raised in our study was that Facebook seems to recruit older age cohorts of family caregivers with younger people using different platforms such as Instagram (Warner et al., 2022). This raises questions about how OSG run through Facebook will respond to changing age demographics. Worryingly, as has been seen in 2023 with Twitter changing to X, corporate entities can change the rules regarding the social media platform, which could dismantle the support arenas for vulnerable groups.

This study contributes further understanding to the language used around engagement, particularly the understanding of participants who have previously been called ‘lurkers’ (e.g. Hurtubise et al., 2019). The findings demonstrate that social support was experienced by this cohort. During the research process, this cohort was named by the participants themselves as 'readers', a term we advocate for use. Many people got value out of only reading content and reframing the language away from lurker, which can have negative connotations, to a term like reader, can help enhance our understanding of how people are generating social support and benefits from OSG. We suggest further research to explore this more nuanced categorization of participants in OSG.

While there are several limitations to the study, many have been discussed in previously published paper (Daynes‐Kearney & Gallagher, 2023a). In particular reference to this study, the Perski et al. (2017) framework relates to digital behaviour change interventions, which have a different focus than OSGs. This study also cannot concretely demonstrate a causal relationship between the types of social support and the elements for engagement and suggests further research would strengthen the connection between these two concepts. Our OSG was also established during the recent COVID‐19 pandemic when other forms of support were removed, and this may have also influenced our findings somewhat, but it is worth noting that our findings mapped very well onto Perski et al. (2017) framework generated pre‐pandemic indicating consistency across time. Finally, as the group was not ethnically diverse, we cannot generalize our findings, but it does point to factors that should be explored further, such as whether there were specific barriers to members of minority groups joining the group.

### New framework: Journey of Engagement and Support in Online Support Groups for Family Caregivers

**FIGURE 1:**
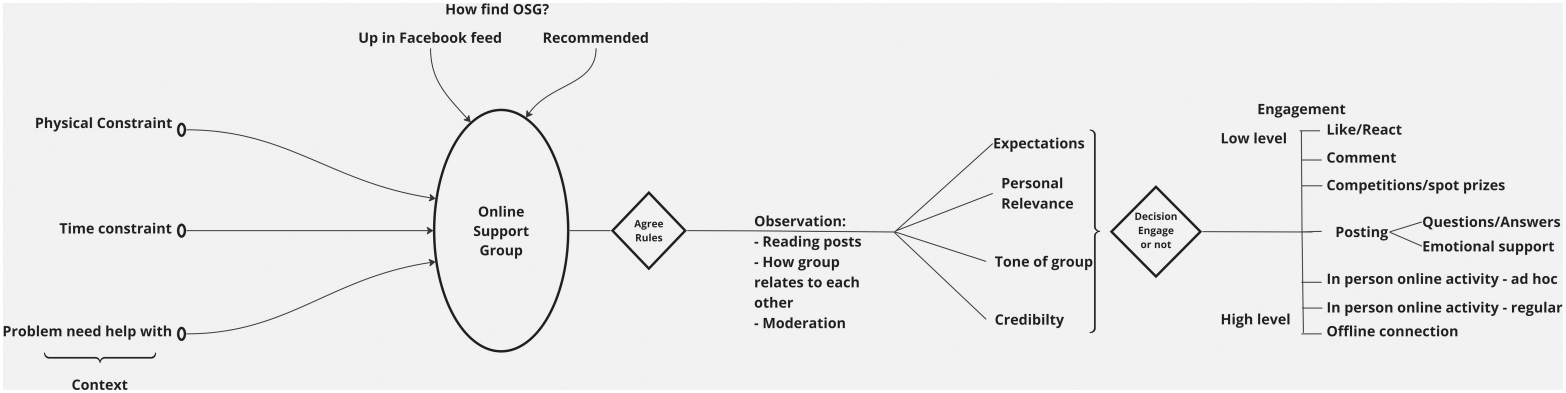
Journey of Engagement and Support in Online Support Groups for Family Caregivers.

Drawing together the findings in response to the research questions, we now present a new framework, the *Journey of Engagement and Support in Online Support Groups for Family Caregivers* (Figure 1). In this framework, the context of the family caregiver involves physical and/or time constraints and has a problem that they need help with. They find an online group, either through social media platforms they are part of, in this case Facebook, or through recommendations. After agreeing to group rules, they may have a period of observation where they read content and monitor the group, assessing key elements such as whether the group meets expectations, is personally relevant and the tone and credibility of the group. During observation, implicit and explicit group norms are understood, such as the role of the moderators, what types of information are shared and how the group responds to emotional distress. They will then decide about engaging with the group and at what level from low to high levels. Social support is experienced and generated at all levels of engagement, regardless of whether the participant only reads, reads and reacts, reads, reacts and comments or takes part in all types of activities and modes of communication on offer. Social network connections formed in the group may lead to offline connections.

## CONCLUSION

Overall, we found that all types of social support had a positive impact on engagement and that the key elements of engagement could encourage or discourage the opportunities for generating social support. There does not appear to be a wide scope of literature focusing on both social support and engagement in OSG for family caregivers and so this study presents new and novel findings about the types of social support generated, key elements of engagement and how these relate to each other. Bringing together the findings from the research, we present a new framework for understanding engagement and social support in OSG for family caregivers. This framework can be used to support practitioners in understanding how family caregivers interact with OSG to better structure their groups to enable maximum support to and engagement by participants. It also contributes to theory in this area and could be used to generate testable hypothesis about the relationship between social support and engagement.

## AUTHOR CONTRIBUTIONS

**Rosemary Daynes‐Kearney:** Conceptualization; investigation; funding acquisition; writing – original draft; methodology; validation; visualization; writing – review and editing; project administration; formal analysis; data curation; resources; software. **Stephen Gallagher:** Conceptualization; investigation; funding acquisition; methodology; validation; visualization; writing – review and editing; formal analysis; project administration; data curation; supervision; resources.

## CONFLICT OF INTEREST STATEMENT

The authors declare there is no conflict of interest with the work.

## Supporting information


Appendix S1

Appendix S2

## Data Availability

Data availability raw data (video recordings and associated raw transcripts) for these data are not publicly available to preserve individuals' privacy under the European General Data Protection Regulation. Transcripts that have been de‐identified are currently being prepared for submission to the University of Limerick repository. Updates to the availability of the dataset will be available on https://osf.io/yqp6n/wiki/Dataset/.
