# Supplementary material for: Online support groups for family caregivers: A qualitative exploration of social support and engagement
Source: Br J Health Psychol. 2024 Nov 7;30(1):e12764. doi: 10.1111/bjhp.12764 (PMC11586818; doi:10.1111/bjhp.12764)
Supplement: Supplementary file 1 — Appendix S1 [file BJHP-30-0-s001.docx]

**Table 1a**

*Codebook based on Cutrona & Suhr (1993) typology of social support*

| Type of social support | Key terms |
| --- | --- |
| Informational | Suggestion/advice; referral; situation appraisal; teaching |
| Emotional | Relationship; physical affection; confidentiality; sympathy; listening; understanding/empathy; encouragement; prayer. |
| Tangible | Lending; direct task related to stressor; indirect task related to stressor; active participation in action to reduce stress; willingness to help. |
| Esteem support | Compliments; validation of recipient’s perspective of situation; relief of blame. |
| Network support | Access to new companions; presence to spend time or be there; companions of similar interests or experience |

**Table 2a**

*Codebook based on Perski et al (2014) framework for engagement in web-based technologies*

| Context | How relates to engagement | Included in analysis |
| --- | --- | --- |
| Population characteristics |  |  |
| Motivation | Positively associated. |  |
| Expectations | Engage more if there is a match between expectations and intervention. | Y |
| Mental health | Poor mental health negatively associated with engagement. | N |
| Experience of well-being | Believing one does not need to work on certain issues negatively associated with engagement. | N |
| Need for cognition | Tendency to process large amounts of information positively associated with engagement. | N |
| Self-efficacy | Positively associated with engagement | Y |
| Personal relevance | Extent to which it applies to individual positively influenced engagement | Y |
| **Demographics** |  | Y |
| Age | Trend towards higher age positively associated with engagement. |  |
| Gender | Trend towards being a woman positively associated with engagement. | Y |
| Education | Trend towards higher education attainment positively associated with engagement. | N |
| Employment | No trend identified. | N |
| Ethnicity | No trend identified. | Y |
| Computer literacy | Confidence positively associated with engagement – no baseline of computer skills. | y |
| Physical characteristics | Baseline weight presence of comorbidities negatively associated with engagement. | N |
| Setting | Hypothesized to influence engagement | y |
| **Context** | **How relates to engagement** | **Included in analysis** |
| Social environment e.g. culture, media and social cues | Hypothesized to influence engagement |  |
| Physical environment e.g. financial and material resources, time pressure, the healthcare system and policy | Hypothesized to influence engagement | Y |
| Time | Hypothesized to positively associated with engagement. | Y |
|  |  | Y |
| Access to hardware | Hypothesized to positively associated with engagement. | N |
| **Content** |  |  |
|  |  | Y |
| Social support features: Features that facilitate the receipt of social support. | Positively influence engagement. |  |
| Reminders | Positively influence engagement. | N |
| **Delivery** |  |  |
|  |  | Y |
| Mode of delivery | Positively influence engagement. |  |
| Professional support features – ability to connect directly with professional for support. | Positively influence engagement. | Y |
| Control features: features to make users feel that they are in control of and free to make choices about how to interact. | Positively influence engagement | N |
| Novelty generated by regular content updated | Positively influence engagement | Y |
| Complexity | Too complex negatively influence engagement. | Y |
| Ease of use | Hypothesised to positively influence engagement. | Y |
| Personalisation or tailoring of content | Hypothesised to positively influence engagement. | Y |
| Interactivity – two way flow of information | Positively influence engagement. | Y |
| Message tone | Hypothesised to positively influence engagement. | Y |
| Narrative | Hypothesised to positively influence engagement. | N |
| Challenge | Hypothesised to positively influence engagement. | N |
| Aesthetics and design | Hypothesised to positively influence engagement. | N |
| Credibility features – feeling of trust | Hypothesised to positively influence engagement. | Y |
| Familiarity | Hypothesised to positively influence engagement. | Y |
| Provision of guidance/tutorials | Hypothesised to positively influence engagement. | Y |
