# Supplementary material for: Online support groups for family caregivers: A qualitative exploration of social support and engagement
Source: Br J Health Psychol. 2024 Nov 7;30(1):e12764. doi: 10.1111/bjhp.12764 (PMC11586818; doi:10.1111/bjhp.12764)
Supplement: Supplementary file 2 — Appendix S2 [file BJHP-30-0-s002.docx]

**Table 1a**

*Demographics of Interview Participants and type of engagement in group*

| Participant | Age Range | Gender | Caring Relationship | Geographic Area | Types of engagement | Frequency of engagement |
| --- | --- | --- | --- | --- | --- | --- |
| 1 | 70-79 | F | Wife | Urban | Reads  Comments  Posts  Activities | Daily |
| 2 | 50-59 | F | Parent | Semi-urban | Reads  Comments  Posts | Frequently |
| 3 | 40-49 | F | Parent | Rural | Reads  Comments  Posts  Activities | Frequently |
| 4 | 50-59 | F | Parent | Urban | Reads  Activities | Daily |
| 5 | 40-49 | M | Bereaved | Rural | Reads  Comments  Posts  Activities | Several times daily |
| 6 | 60-69 | F | Parent | Rural | Reads  Comments  Posts  Activities | Several times daily |
| 7 | 60-69 | F | Sister-in-law | Rural | Reads  Comments  Posts  Activities | Several times daily |
| 8 | 50-59 | F | Wife and mother | Rural | Reads  Comments  Posts  Activities | Several times daily |
| 9 | 50-59 | F | Daughter | Semi-urban | Reads  Comments  Posts | Several times daily |
| 10 | 40-49 | F | Parent | Urban | Reads  Comments  Posts  Activities | Several times daily |
| 11 | 50-59 | F | Wife | Semi-rural | Reads  Comments  Posts  Activities | Several times daily |
| 12 | 40-49 | F | Parent | Semi-urban | Reads  Comments  Posts  Activities | Several times daily |
| 13 | 50-59 | F | Parent | Urban | Reads  Comments | Several times daily |
| 14 | 40-49 | F | Parent | Rural | Reads  Comments  Posts  Activities |  |
| 15 | 50-59 | F | Parent | Urban | Reads  Comments  Posts | Frequently |
| 16 | 40-49 | M | Parent | Rural | Reads  Comments  Posts  Activities | Daily |
| 17 | 60-69 | F | Sibling | Rural | Reads  Comments  Posts | Infrequently |
| 18 | 30-39 | F | Parent | Urban | Reads  Comments  Posts  Activities | Several times daily |

**Table 2a**

*Types of social support present in the group*

| Type of social support | Present | How Presented | Impact on Engagement | Quotes |
| --- | --- | --- | --- | --- |
| Information | 17/18 | Helpful information on main page and in comments | Positive – people found answers to their questions | *It’s like a walking encyclopaedia.*  Participant 1 |
|  |  | Diversity of information | Positive – people found useful tips from advice and suggestions from carers in different caring situations. | *Little nuggets of information can always be found –*  Participant 10 |
|  |  | High quality information | Positive – people trusted the information they found. | *There's already some much information there, so like that I can't think of an occasion when I did ask a question myself 'cause the information stays there, seems to be a lot up there.*  Participant 3 |
|  |  | Mixed quality information in comments | Could have negative impact by people getting inaccurate information. | *I think the moderator should check stuff at times to see is it genuinely right.*  Participant 15 |
| Emotional | 15/18 | Honest self-disclosures | Positive – people felt their emotional burden was relieved. Readers recognised themselves in posts from others. | *It can even just be like I'm going a bit mental here, you know, going mad and I just wanted to vent.*  Participant 2 |
|  |  | Sharing positives and challenges | Positive – people enjoyed sharing good news and being supported through difficult times. | *It's grand that I can rant on that to other people, but then I've also got on it like whenever I’ve had a really good day or something nice to happen.*  Participant 7 |
|  |  | Use of emojis and pictures | Positive – allowed people to show support without words. | *A hug, you know, then the little heart, you know, the hug symbol.*  Participant 18 |
|  |  | Encouragement and empathic statements in response to posts | Positive – people engaged with the poster in response to content in the post. | *I don't reply to all the comments, but sometimes someone will put something in and I go. Oh you poor old thing and I sent a comment on to them, you know and say well hopefully that made them feel better. Participant 17.* |
| Esteem | 8/18 | Affirmation to poster that they were doing their best. | Positive – poster felt relieved of emotions such as guilt or failure | *It kinda was self-validation…increased your self-worth, you know so it was nice*.  Participant 16 |
|  |  | Affirmation to participants that they were providing a valuable role. | Positive – people felt valued and visible | *People are putting up really nice things and you know. I think people are just trying to build each other up.*  Participant 4 |
|  |  | Positive non-judgemental responses to self-disclosures. | Positive – people felt safe and relieved of stigma | *I think my first coffee morning I was very nervous. I didn't know what to expect… the support is unreal. There's no judgment.*  Participant 12 |
|  |  | Acknowledgement of valuable contribution of sharing knowledge and skills. | Positive – people felt valued and visible | *The life experience that I have is as a carer is valuable…I can use it to help someone else or pass on my information or pass on my experience and it might help someone else.*  Participant 11 |
| Social Network | 13/18 | Sense of feeling connected to large group of people in similar situations. | Positive – people had more social connections through the group | *I have made friends…yes they are virtual friends and we’ll probably never meet, but it’s that support that you can actually give to a stranger.*  Participant 14. |
|  |  | Removal of geographical barriers to enable the building of connections others. | Positive – people connected with others they wouldn’t have met locally | *Even though you’d have people from all over the country, you're engaging with them, and you're talking to them.* Participant 1 |
|  |  | Removal of time boundaries to enable the creation of connections with others. | Positive – people were able to connect to others at times that suited them | *It’s like having a best friend on call 24/7*  Participant 9 |
| Tangible | 13/18 | People received useful resources from the organisation. | Positive – these resources directly and indirectly helped them in their caring role. | *They sent us out a carers book…with all the different entitlements* Participant 6 |
|  |  | People received gifts from the organisation. | Positive – these gifts made people feel valued and visible. | *Sweets and stuff like that… It's great to get anything like that. It's lovely.*  Participant 6 |
|  |  | Items were advertised and passed on through the group. | Positive – this provided direct support to the participants | *If they had stuff in the house that would be useful to another carer…they put it up for saying that they’ll give it away… I find that's very useful.*  Participant 6. |

**Table 4a**

*Contextual elements, Engagement and Social Support in the group*

| Elements | Sub elements | Impact on Engagement | How Related to social support | Quotes |
| --- | --- | --- | --- | --- |
| Constraints to physical environment and time | Group responded to Covid-19 lockdowns and withdrawal of services | Positive - provided an outlet for carers when no others existed | Provided all types of social support | *Covid was a very lonely time for carers.*  Participant 14 |
|  | Flexibility and availability at any time | Positive – could engage when able to | Facilitated easy provision and receipt of social support | *Care sometimes can be 24/7. So you can have that space where maybe your loved one is maybe resting, or you could have another family member sitting with them and it's an activity that you could do to give you a break from your role as a carer.*  Participant 5 |
| Expectations | Clear understanding of group purpose | Mixed – maintains engagement if understood what group was about, but negative if unclear e.g. name support group can mean different things. | Important for group maintenance for participants continue to avail of opportunities for support | *Maybe it isn't a supportive group…maybe it's just a place to share stuff that's going on, online. To me, that's what it is. That's what I use it for now.*  Participant 18 |
|  | Experience congruent with expectations | Positive – influenced decision about getting involved and how deep involvement would be in group | Important for group maintenance for participants continue to avail of opportunities for support | *It doesn’t really matter who you are or what place you’re at, it’s a very supportive group.*  Participant 2. |
| Personal relevance | Group content and members were relevant to participant. | Positive - Comfort with people in similar situations | Provided all types of social support | *I found so much information, so much support. It's been unreal.*  Participant 12 |
| Personal relevance | Social comparison | Mixed – can dis-courage engagement if feel others need more support | Can reduce opportunities to provide or receive social support | *To be honest I do feel guilty sometimes because I look at some of what people have in terms of caring… I felt that I really shouldn't be here, getting a spot prize because…I have nothing compared to that lady.*  Participant 17 |
|  |  |  |  |  |
| Existing social support | Other places to get social support | Negative – if had other avenues affected frequency and type of engagement | Can reduce opportunities to provide or receive social support in the group | *I look in every day but I don't actually involve myself everyday, you know, because I know some people need it and it's probably the only the only source outside of the outside world like they would have. But I don't 'cause I'm very lucky you know so I don't.*  Participant 7 |
|  |  |  |  |  |
|  |  |  |  |  |
|  |  |  |  |  |
|  |  |  |  |  |

**Table 5a**

*Content elements and Engagement in the group*

| Elements | Sub elements | Impact on Engagement | Related to social support | Quotes |
| --- | --- | --- | --- | --- |
| Social Support Features | Direct messaging | Positive – enabled participants to ask questions or post anonymously | Facilitated easy provision and receipt of social support | *Then last night the girl that put up that comment on that page went on with her name and said look, it's me. I just wanted to say thanks I didn't have the balls to put my name up, but I do now…I'm not alone …that was great. ­*  Participant 12. |
|  | Variety of communication features | Positive – could engage how most comfortable | Facilitated easy provision and receipt of social support | *I find it hard to find the words to communicate…it's easier to type it. Instead of voice it, so that's another reason why that group is so important.*  Participant 8 |
|  | Timely response by moderators | Positive – build trust with participant | Key for informational and emotional support | *I have…private messaging with the staff and those who organized the page. They've always been very good in responding.*  Participant 5 |
| Activities | Variety of activities | Positive – something for everyone | Provides opportunities to develop social network support | *Activities and online courses and programs and social meetups…there was a lot going on… it's just going from strength to strength.*  Participant 5 |
|  | Developed in response to expressed need in the group. | Positive | Important for group maintenance to continue to avail of opportunities for support | *The team there kind of evolved with it and they got a feel for what was needed they were very reactive to what people wanted.*  Participant 3. |
| Activities | No expectations on participant | Positive – relieved pressure on participants and encouraged repeat attendance. | Important for group maintenance to continue to avail of opportunities for support | *No expectations compared to normal book group.*  Participant 3. |
| Activities | Unclear how to participate | Negative – prevented from joining or continuing with group | Can reduce opportunities to provide or receive social support | *I was ashamed of my life then to look for another book the last time because…I didn't know if they were going to going to have a review with the books afterwards.*  Participant 7 |
|  |  |  |  |  |
|  | Had to participate in real time | Negative – not always suitable time for participants | Can reduce opportunities to provide or receive social support but important for social network support | *I think a lot of like the interactions are in the evening time…I don't get a lot of downtime and in the evening time that's just our busy time.*  Participant 18 |
|  | New members joining established groups | Negative – familiarity made it difficult for new people to take part in group | Can reduce opportunities to provide or receive social support | *Not one person said hello to me, which I thought was odd, you know, and they're all talking to each other and all laughing and joking.*  Participant 4 |
|  |  |  |  |  |
| Incentives for engagement | Spot prizes, competitions, gifts and targeted events | Positive – Fun way to be involved and build community. | Builds all types of support | *You’d be surprised how somebody liking your photograph can make you feel better about yourself.*  Participant 16 |
|  | Immediacy of some incentives | Mixed – not everyone able to respond quickly due to caring responsibilities. | Can reduce opportunities to provide or receive social support | *It made me feel a little bit sad because. It just showed me how busy my life is that I'm not even able to go online* .  Participant 18. |
| Incentives for engagement | Personal messages and ‘nudges’ | Positive – welcome and encourage participant in the group | Key for development of emotional, esteem and social network support | *When I joined the group first…one of the moderators…he told me to keep on, because male carers are scarce, there are very few who interact and he said that’ll only encourage other male carers too…it kind of makes you feel special.*  Participant 16 |
|  | Group getting bigger | Mixed – concern over how to keep personal touches and sense of community with large group | Can reduce opportunities to provide or receive social support | *Just to be conscious at the group is going so big and you know not to kind of lose people out there*. *In scaling it, it kind of its the personal touch that I would be what I would be worried with would disappear somewhat.*  Participant 3 |

Table 7a

Delivery elements and Engagement in the group.

| Elements | Sub-elements | Impact on Engagement | How related to social support | Quotes |
| --- | --- | --- | --- | --- |
| Mode of delivery and ease of use | Use of Facebook | Positive – not geographically bound, easy to find, part of day-to-day life. | Facilitated easy provision and receipt of social support | *The carers group is more accessible on Facebook. More people see…and interact with it.*  Participant 8. |
|  |  | Mixed – used by a certain age group. |  | *Not a whole pile of young people would use Facebook… Facebook would have an older audience nearly.*  Participant 15 |
|  | Use of Videoconferencing | Mixed – enabled face-to-face real time interactions but technology was a barrier to some. | Key for development of social network support | *You could connect with other carers in that virtual sphere or virtual worlds and you could actually physically see them and interact with them that was great…for connection.*  Participant 5  *There’s so much going on in daily life, my stress levels are already so high that I couldn’t actually cope with that.* Participant 14 |
| Professional support and interactivity | Trained moderators | Positive – participants feel group is managed well | Facilitated easy provision and receipt of social support | *There’s people there doing a job of monitoring it properly. So it's well set up…it's well looked after.*  Participant 13 |
|  | Two-way flow of communication | Positive – builds relationships | Facilitated easy provision and receipt of social support | *If you have any problems you just… send a message to them… they will answer you and you know that that support is there. You know the backup is there.*  Participant 1 |
| Professional support and interactivity | Welcoming new members | Negative if new members not welcomed to group | Key for development of emotional and esteem support | *The organizer should have at least said well this is a new member of the group and welcome to our quiz or whatever… I'll see how it goes the next time and if I get the same reaction well then that's it.*  Participant 4 |
|  | Responsiveness | Positive – participants feel that their input is important | Key for informational and emotional support | *If they can’t do anything for you, they will go and get the information and come back to you…they will try to get the answers for you.*  Participant 1 |
|  | Dominant members | Negative – can take over group and mean activities don’t meet expectations | Can reduce opportunities to provide or receive social support | *The gardening club can go a little bit off kilter at times, it doesn’t all be gardening –* Participant 4 |
|  | Moderator biographies | Mixed – can help participants to know any caring background of moderators. | Can contribute to social network support | *You don't know their particular situations. So it's hard to know if they’re carers themselves or not. It does have a little bit of an impact*.  Participant 3 |
| Tone of group | Friendly and fun | Positive – Fun way to be involved and build community. | Important for group maintenance and opportunities for support | *In other Facebook groups…there's always this element of judgment or snippiness…there is none of that in the Carer Alliance group.*  Participant 11 |
|  | Non-judgemental | Positive – encourages people to share and seek support. | Key for emotional and esteem support | *There’s a lot of care going on in the Care Alliance world…it’s the care coming forward.*  Participant 14. |
|  | Modelling group rules and norms | Positive – welcome and encourage participant in the group. | Important for group maintenance to continue to avail of opportunities for support | *There can be some judgmental people on it, like on everything, but they're quickly shut down on it, it's very positive.*  Participant 8 |
|  | Problems dealt with quickly and respectfully | Positive – people see group as positive place. | Important for group maintenance to continue to avail of opportunities for support | *If someone puts up a comment that maybe people feel that's a little bit harsh. People are inclined to say, you know, that's not really nice without being hard on the person.*  Participant 11 |
|  |  |  |  |  |
| Credibility | Safety created by group rules being maintained | Positive – people feel that group is safe to be part of and use group. | Key for development of emotional and esteem support | *You can share something to the group, but it doesn't go directly straight up onto the page and moderators has to look at it and see. Is it appropriate to be shared?*  Participant 5 |
|  |  |  |  |  |
|  | Privacy – what is said in the group stays in the group | Positive – encourages people to share things that they wouldn’t otherwise | Key for development of emotional and esteem support | [*Privacy is] very important because you wouldn't be as open and you wouldn't be as honest, you would filter everything… It's even better to know that it stays within the group because there's an awful lot of personal things that you wouldn't say to family members or wouldn't say on the street or to other, to friends because they're not going to get it*.  Participant 9 |
|  |  |  |  |  |
|  | Privacy enabled by anonymous posting. | Positive - encourages people to share things that they wouldn’t otherwise. | Key for development of emotional and esteem support | *It's great. It really is like because some things would be you'd feel bad saying them… So it's just it's that extra little layer of privacy for you… you're still getting the support that you need. It's just people don't know who they're actually supporting.*  Participant 10 |
|  | Trust developed by moderator consistency, high quality information, effective communication on decisions | Positive – participants understand process behind decision making. | Key for development of emotional and esteem support | *He declined. And he said do you mind, if I don't put that up…and I said no, that's fine*  Participant 13 |
